# Cell-Free Supernatants (CFSs) from the Culture of *Bacillus subtilis* Inhibit *Pseudomonas* sp. Biofilm Formation

**DOI:** 10.3390/microorganisms10112105

**Published:** 2022-10-24

**Authors:** Shirmin Islam, Md. Liton Mahmud, Waleed H. Almalki, Suvro Biswas, Md. Ariful Islam, Md. Golam Mortuza, Mohammad Akbar Hossain, Md. Akhtar-E Ekram, Md. Salah Uddin, Shahriar Zaman, Md. Abu Saleh

**Affiliations:** 1 Microbiology Laboratory, Department of Genetic Engineering and Biotechnology, University of Rajshahi, Rajshahi 6205, Bangladesh; 2 Department of Pharmacology and Toxicology, Faculty of Medicine, Umm Al-Qura University, Makkah 21955, Saudi Arabia; 3 Department of Science and Humanities, Bangladesh Army International University of Science and Technology, Cumilla 3500, Bangladesh; 4 Department of Pharmacology and Toxicology, Faculty of Medicine in Al-Qunfudah, Umm Al-Qura University, Makkah 21955, Saudi Arabia

**Keywords:** *Bacillus subtilis*, *Pseudomonas* sp., biofilm inhibition, cell-free supernatant

## Abstract

Biofilm inhibition has been identified as a novel drug target for the development of broad-spectrum antibiotics to combat infections caused by drug-resistant bacteria. Although several plant-based compounds have been reported to have anti-biofilm properties, research on the anti-biofilm properties of bacterial bioactive compounds has been sparse. In this study, the efficacy of compounds from a cell-free supernatant of *Bacillus subtilis* against a biofilm formation of *Pseudomonas* sp. was studied through in vitro, in vivo and in silico studies. Here, in well diffusion method, *Bacillus subtilis* demonstrated antibacterial activity, and more than 50% biofilm inhibition activity against *Pseudomonas* sp. was exhibited through in vitro studies. Moreover, molecular docking and molecular dynamics (MD) simulation gave insights into the possible mode of action of the bacterial volatile compounds identified through GC-MS to inhibit the biofilm-formation protein (PDB ID: 7M1M) of *Pseudomonas* sp. The binding energy revealed from docking studies ranged from −2.3 to −7.0 kcal mol^−1^. Moreover, 1-(9H-Fluoren-2-yl)-2-(1-phenyl-1H-ttetrazole5-ylsulfanyl)-ethanone was found to be the best-docked compound through ADMET and pharmacokinetic properties. Furthermore, MD simulations further supported the in vitro studies and formed a stable complex with the tested protein. Thus, this study gives an insight into the development of new antibiotics to combat multi-drug-resistant bacteria.

## 1. Introduction

Antibiotics are weapons to combat infectious microbes. From the period of the 1930s to the 1960s, the development of antibiotics peaked [1]. Unfortunately, due to the emergence of pathogens that were resistant to antibiotics, researchers were unable to keep up the pace of antibiotic discovery, and this era came to an end. According to the WHO (2019), nearly 700,000 people die each year as a result of infections caused by drug-resistant microbes. If nothing is done, it is expected to become a major cause of mortality by 2050, even overtaking cancer [2]. The predisposing factors linked to the emergence of antibiotic resistance include the persistent failure to discover new antibiotics and the careless use of antibiotics [3]. As a result, the global health sector is facing the problem of antibiotic resistance; moreover, the situation is getting worse due to the rise of resistant bacterial strains [4]. Antibiotic resistance develops as an adaptation mechanism to antibiotic exposure. Biofilms are surface-attached communities of microbial cells encased in an extracellular matrix that are much more resistant to antimicrobial agents than non-adherent, planktonic cells. As a result, biofilm-based infections are extremely hard to treat [5]. The resistant nature of biofilm is a matter of great concern for the global health care system and many studies have been conducted to explore novel, natural, and effective anti-biofilm agents [6]. Pathogenic bacteria frequently form biofilms, which increases their resistance to antimicrobials and exacerbates the issue [7]. Prominent biofilm-forming strains include *Vibrio cholerae*, *Pseudomonas aeruginosa* [8], *Staphylococcus aureus* [9], and *Acinetobacter baumannii* [10]. As biofilm formation is one of the methods by which bacteria develop resistance by chemical signaling, targeting the biofilm-related proteins or deciphering the biofilm signaling pathway will help to discover antimicrobial agents [6]. Nature is the greatest source of bioactive compounds, which can be used to develop anti-biofilm agents. Marine organisms, plants, and microorganisms are an unfathomably large source of anti-biofilm agents [11]. Even so, insufficient productivity and biosynthetic difficulty restrict the production of naturally synthesized compounds. Over the last few years, bioinformatics tools have been using to develop drugs against several diseases. This not only saves time but also minimizes the experimental cost, while moreover creating a broad sector for conducting versatile research [12].

The bioactivity of volatile organic compounds produced by bacteria is effective against pathogenic bacteria. Among other chemical classes, these volatile compounds are small molecules that are biosynthesized by primary and secondary metabolic pathways including sulfur, alcohols, aliphatic and aromatic hydrocarbons, terpenes, nitrogen, and compounds. Volatile organic compounds produced by the bacteria aid in intra- or intercellular communication and provide protection from other microbes. Given this, it is possible to view volatile bacterial compounds as a highly promising source of medications with antibacterial bioactivity [13]. 

In this study, we explored the anti-biofilm activity of *Bacillus subtilis* against *Pseudomonas* sp. The anti-biofilm studies were performed in microtiter plate and was validated using light scanning electron microscopy. For in silico work, volatile compounds were docked against a caseinolytic protease (7M1M) protein target to study their interactions because this plays a crucial role in biofilm formation of *Pseudomonas* sp. [14]. Molecular dynamics (MD) simulation studies were carried out to explore the stability of the docked complex.

## 2. Materials and Methods

### 2.1. Bacterial Strain Selection

*Bacillus subtilis* and four other selected strains were collected from the Microbiology Laboratory, Department of Genetic Engineering and Biotechnology, University of Rajshahi, Bangladesh. 

### 2.2. In Vitro Antagonistic Test

The antibacterial activity of the *Bacillus subtilis* against the selected bacterial strains (Table S1) was checked using the agar well diffusion method with some modifications [15]. Briefly, MH (Mueller Hinton) agar plates were prepared and a suspension of the selected bacterial strains (Table S1) previously cultured in LB liquid was spread on the plate; then, after 5 min of air drying, 5mm wells were made in the agar plates and filled with 100 µL CFS of the *Bacillus subtilis*. After this, the plates were incubated at 37 °C for 24–48 h. 

### 2.3. Biofilm-Formation Activity

The biofilm formation ability of *Pseudomonas* sp. was examined according to the method described previously on 96-well microtiter plates [16]. Put simply, selected overnight-cultured bacterial strains were allowed to grow on the wells of a microtiter plate (Tarsons, India) and incubated in an incubator at 37 °C for twenty-four hours. After the incubation period, the plate was rinsed twice with double distilled water and stained with crystal violet (0.1%). After 60 min, the glass slide was rinsed twice with phosphate buffer and allowed to dry for around 1 h. The crystal violet was dissolved by rinsing it, and the OD595 was recorded using microplate reader (Bio-Rad, Hercules, California, USA). The OD values were considered the biofilm-formation level for each strain. Finally, the pathogen with the highest biofilm-formation potential was used for anti-biofilm assays. [17].

### 2.4. Inhibition of Biofilm Formation by CFSs

The anti-biofilm activity of the CFSs of *Bacillus subtilis* on the biofilm-formation ability of *Pseudomonas* sp. was examined using the protocol of Ray et al. [18]. Here, 100 µL CFSs (centrifuged 10,000× *g* rpm/8 min at 4 °C to obtain cell free supernatants) was added with the bacterial inoculum (100 µL). The disruption percentage was calculated by the following equation:

Disruption Percentage = (ODcontrol − ODsample) × 100/ODcontrol

### 2.5. Scanning Electronic Microscope (SEM)

The presence of *Pseudomonas* sp. and its biofilm was detected by SEM following the previously described method with some modifications [19]. Briefly, the previously described biofilm-formation protocol was used to form biofilm on a 96-microtiter plate. After this, the stained film layers were visualized through SEM.

### 2.6. Volatile Compound Analysis through GC-MS

The volatile compounds of *Bacillus subtilis* was analyzed using gas chromatography–mass spectrophotometry (GC–MS) to identify the anti-biofilm compounds. The experimental settings were developed based on previous investigations following the procedure of [20] with some modifications. Peaks were identified using a spectrum library (NIST 14.0 library). 

### 2.7. Molecular Identification of Anti-Biofilm Compounds

The potential compounds with anti-biofilm activity from the volatile compounds of *Bacillus subtilis* were examined by molecular docking using AutoDock Vina [21]. The 3D structure of the compounds was downloaded from the PubChem database. The 3D crystal structure of the nucleosidase (MTAN) enzyme of *Pseudomonas* sp. (7M1M) of the receptor protein was then downloaded from Protein Data Bank. The analysis of docked complexes was performed using PyRx, PyMol, LigPlot+, and Discovery Studio [2].

### 2.8. Pharmacokinetic Properties Analysis

SwissADME, AdmetSAR, and pkCSM server were used for the prediction of the pharmacokinetic properties of volatile compounds [22,23]. The main focus of this study, including absorption, distribution, metabolism, excretion, solubility, toxicity, carcinogenicity, bioavailability, and drug likeliness, fell mainly in this section [24].

### 2.9. Molecular Dynamics Simulation

The molecular dynamics simulation was carried out in YASARA software [25,26] with the assistance of the AMBER14 force field [27,28]. Initial optimization, cleaning, and hydrogen-bond orientation were performed on docked complexes. A cubic simulation cell was constructed using the TIP3P solvation model with periodic boundary conditions [29,30,31]. Besides this, the simulation cell was stretched by 20 Å in each direction beyond the protein-ligand complexes. The physiological condition of the simulation cell was adjusted as pH 7.4, 298 K, and 0.9% NaCl. The steepest gradient algorithm (5000 cycles) was used for preliminary energy minimization using the simulated annealing method and the time step for the simulation cell was then set as 1.25 fs [32,33]. Additionally, the long-range electrostatic interactions were computed by using the Particle Mesh Ewald (PME) system and an 8.0 Å cutoff radius [34,35,36,37]. The simulation was run for 100 ns at a constant temperature and pressure, along with the Berendsen thermostat, and the data of the simulation trajectories were recorded after every 100 ps [38,39]. The data of the simulation trajectories were utilized after the simulation process for the analysis of the root mean square deviation (RMSD), radius of gyration (Rg), root mean square fluctuation (RMSF), solvent accessible surface area (SASA), and hydrogen bond [40,41,42]. 

### 2.10. Statistical Analysis

The experiment was performed with three replications of each biological sample. All values were reported as the mean ± SEM (standard error of the mean). The DMRT (Duncan’s Multiple Range Test) was used to analyze the significance of each group’s data at a *p* ≤ 0.05 level of significance in a one-way ANOVA in SPSS Statistics 26 software. Graph Pad Prism 8.0.2.263 was used for preparing all figures.

## 3. Results

### 3.1. In Vitro Antagonistic Test

In vitro antagonism studies using the well-diffusion method produced a positive result, with an inhibitory zone visible encircling the macro-colonies (Table 1). *Bacillus subtilis* had average (18.33 ± 1.0 mm) inhibition zone against *Pseudomonas* sp., indicating that the bacteria can produce antibiotics. Moreover, for the other three strains, no inhibition potentiality was observed.

### 3.2. Biofilm-Inhibition Assay

**Figure 1:**
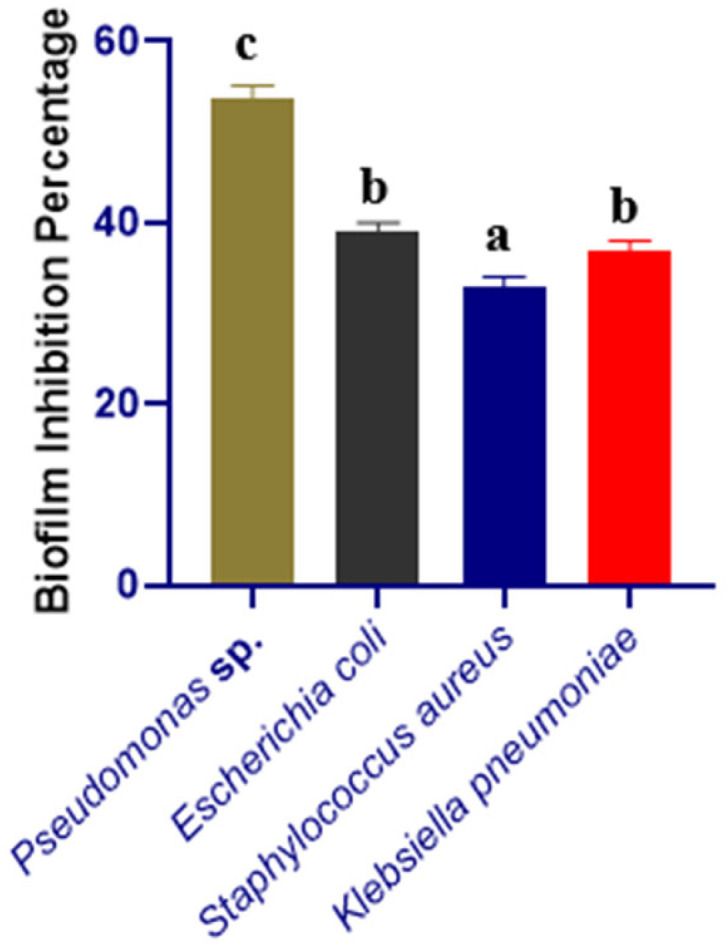
Biofilm-inhibition percentage of *Klebsiella pneumoniae*, *Pseudomonas* sp., *Escherichia coli*, *Staphylococcus aureus* by the CFS of *Bacillus subtilis*. Different letters indicate significant differences between mean ± SD of replications (*n* = 3) at a *p* < 0.05 significance level.

**Figure 2:**
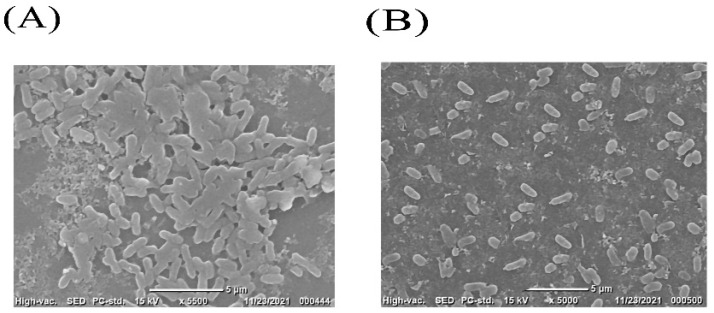
Representative scanning electron micrographs showing biofilm disruption on glass surfaces (**A**) Biofilm formed by *Pseudomonas* sp. (**B**) biofilm disruption by co-incubation with 100 µL of CFS from *Bacillus subtilis* and bacterial culture of *Pseudomonas* sp.

All of the selected pathogenic bacterial strains demonstrated robust biofilm-formation abilities in 96 microtiter plate assays. Selected strains were independently co-cultured with CFSs of *Bacillus subtilis*, and the effect of the co-incubation on the biofilms is shown in Figure 1. When *Pseudomonas* sp. was co-cultured with CFSs of *Bacillus subtilis*, the greatest biofilm inhibition (54%) was seen. This outcome was also supported by SEM images (Figure 2). Some *Pseudomonas* sp. cells were shown to be deformed after treatment with CFSs of *B. subtilis* (Figure 2B).

### 3.3. Volatile Compound Analysis by GC-MS

**Figure 3:**
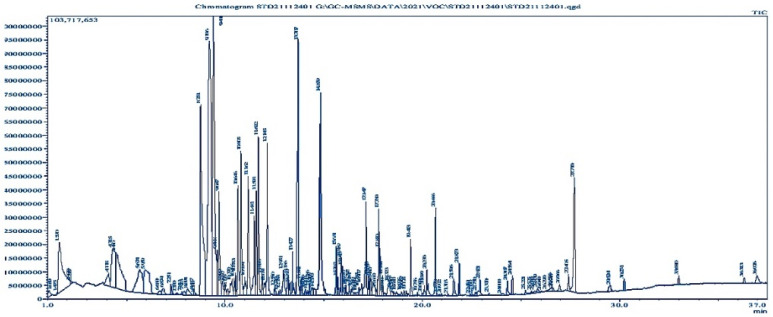
GC-MS chromatogram showing various bioactive compounds of *Bacillus subtilis*.

It was found that *Bacillus subtilis* contains several phenolic, -OH, and unsaturated carbonyl compounds. The biological identity of 85 compounds from *Bacillus subtilis* was validated by interpreting the mass spectra of GC-MS (Figure 3) of the unknown components and comparing them with the database kept in the National Institute Standard and Technology (NIST) library. Name, retention time, and peak area of the compounds were determined. Comparing each component’s average peak area to the total area allowed us to determine the amount of each component in terms of its proportional percentage (Table S1).

### 3.4. Molecular Docking Analysis

**Figure 4:**
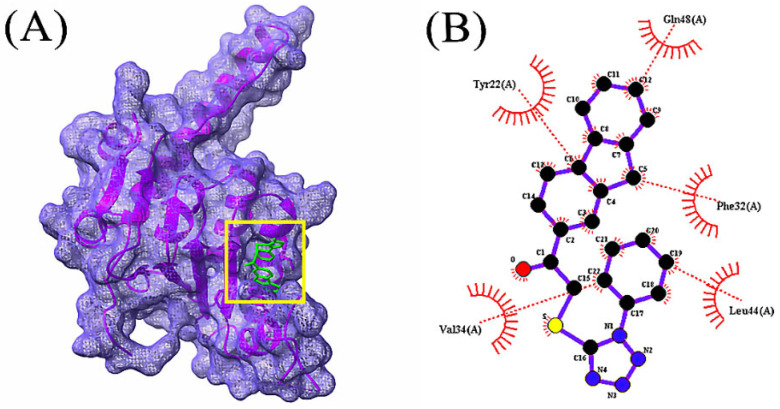
Docking simulation between protein-ligand interactions of caseinolytic protease (Clp) enzymes *Bacillus subtilis* (PDB ID: 7M1M) and 1-(9H-Fluoren-2-yl)-2-(1-phenyl-1H-tetrazol-5-ylsulfanyl)-ethanone, where (**A**) Surface view using Chimera (binding pocket), (**B**) 2D view using LigPlot+.

**Figure 5:**
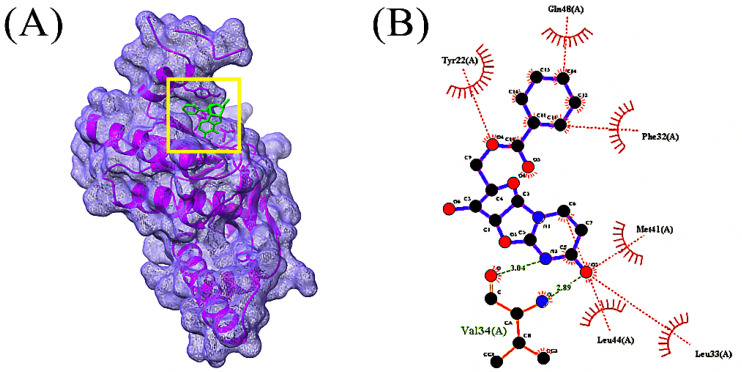
Docking simulation between protein-ligand interactions of caseinolytic proteases (Clp) enzymes *Bacillus subtilis* (PDB ID: 7M1M) and oxalic acid, cyclohexylmethyl tridecyl ester, where (**A**) Surface view using Chimera (binding pocket), (**B**) 2D view using LigPlot+.

**Figure 6:**
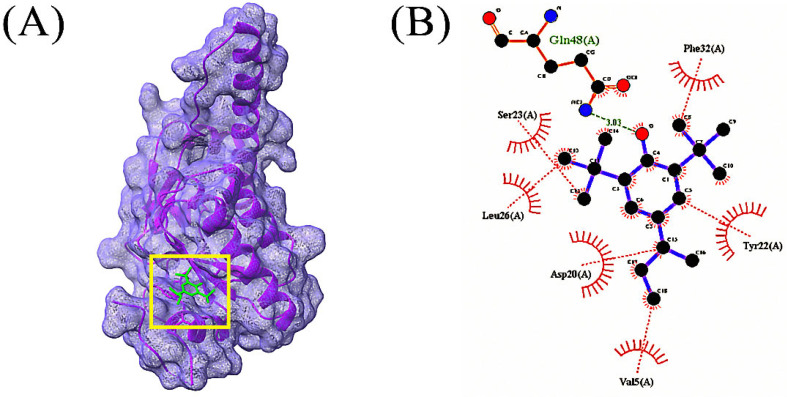
Docking simulation between protein-ligand interactions of caseinolytic proteases (Clp) enzymes *Bacillus subtilis* (PDB ID: 7M1M) and bis(pentamethylcyclotrisiloxy)tetramethyldisiloxane, where (**A**) Surface view using Chimera (binding pocket), (**B**) 2D view using LigPlot+.

**Figure 7:**
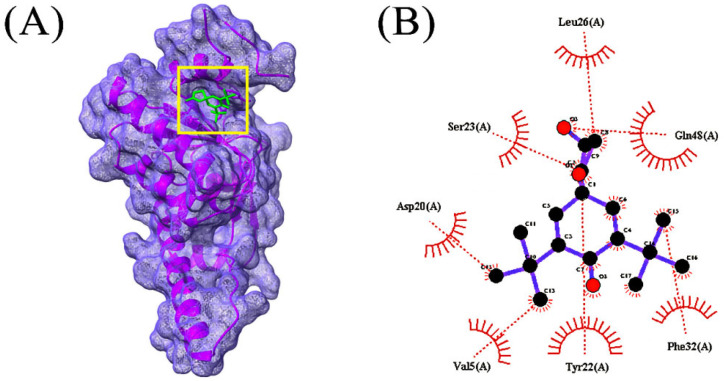
Docking simulation between protein-ligand interactions of Caseinolytic proteases (Clp) enzymes *Bacillus subtilis* (PDB ID: 7M1M) and 2,2’-anhydro-1-arabinofuranosyluracil, where (**A**) Surface view using Chimera (binding pocket), (**B**) 2D view using LigPlot+.

In total, 85 volatile compounds were docked against the *Bacillus subtilis* caseinolytic protease (Clp) enzyme (PDB ID: 7M1M). The highest docking score was −7.0 kcal/mol and the lowest score was −2.3 kcal/mol. A total of 30 volatile compounds’ docking scores were more than −5 (Table S2). Out of 85 volatile substances, the top 10 had docking scores ranging from −7.0 to −5.6 kcal/mol. Four volatile substances exhibited docking scores of more than −6.0 kcal/mol, and the values of those scores and the interactions between protein ligands are shown in Table 2, Figure 4, Figure 5, Figure 6 and Figure 7, and Table S3, respectively.

### 3.5. ADMET Prediction

Molecular weight, number of H-bond acceptors, number of H-bond donors, lop P, log S, TPSA BBB permeability, and human intestinal absorption of the top four compounds from the ADMET investigation are shown in Table 3. The results showed that all compounds, with the exception of bis(pentamethylcyclotrisiloxy) tetramethyldisiloxane, had molecular weights of less than 500 Da. The topological polar surface area (TPSA) score must fall between 0 and 140 in order to qualify as a drug candidate [43]. The TPSA values in those compounds ranged from 52.82 to 99.88. Additionally, none of the four compounds could circumvent the BBB and all four had high absorption capacities.

### 3.6. Molecular Dynamics Simulation

**Figure 8:**
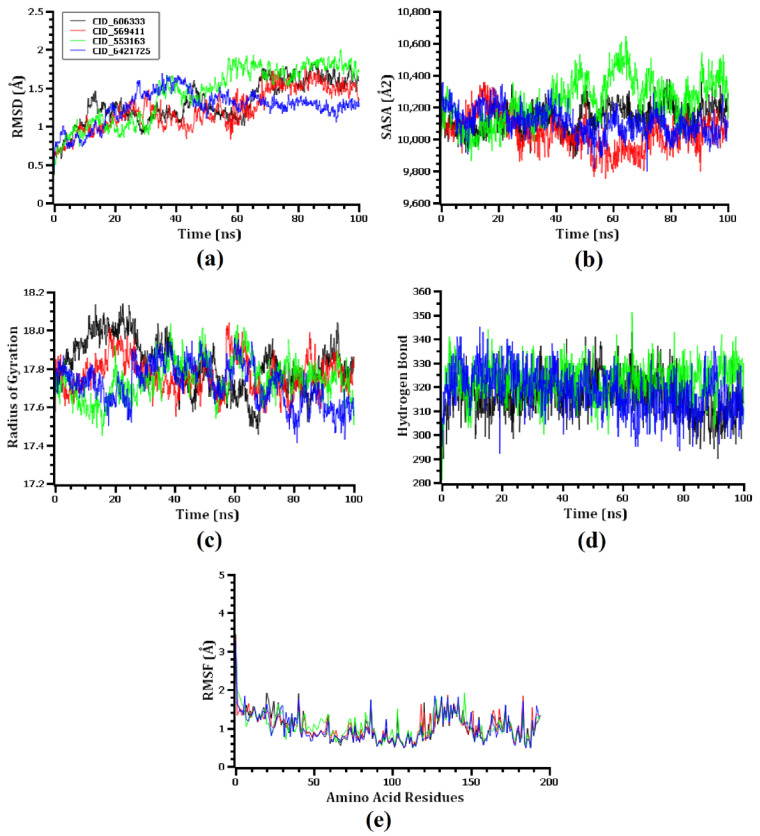
The molecular dynamics simulation study of the top four ligand molecules and 7M1M protein complex, here, (**a**–**e**) designates the RMSD, SASA, Rg, H-bond, and RMSF analysis of the complexes. Here, black color indicating CID 606333, red color indicating CID 569411, green color indicating CID 553163 and blue color indicating CID 6421725.

A molecular dynamics simulation was accomplished in order to investigate the structural stiffness and corroborate the docking scenarios for the top four protein-ligand complexes. In order to explain the stability changes in protein-ligand complexes based on simulated trajectory outcomes, RMSDs of C-alpha atoms were investigated. The protein-ligand complexes including the 1-(9H-Fluoren-2-yl)-2-(1-phenyl-1H-tetrazol-5-ylsulfanyl)-ethanone (CID_606333), 2,2’-anhydro-1-arabinofuranosyluracil (CID_569411), bis(pentamethylcyclotrisiloxy)tetramethyldisiloxane (CID_553163), and oxalic acid, cyclohexylmethyl tridecyl ester (CID_6421725) compounds exhibited an initial RMSD increase because of their instability, as shown in Figure 8a. Among the four complexes, the CID_553163-7M1M complex demonstrated a greater average rise in the RMSD values. On the other hand, the CID 6421725-7M1M complex displayed a lower average RMSD value than the other three complexes. At around 30 ns, the RMSD value of the CID_606333-7M1M complex dropped significantly, then it steadied at about 70 ns and remained stable with very slight variations for the final 30 ns of the simulation time. The CID 553163-7M1M complex has a somewhat higher RMSD profile than the other three complexes, at 50–70 ns, which may explain their increased flexibility. Before 65 ns, the four complexes showed some upward and inward RMSD values in the simulation, but they continued steadily for the final 35 ns with negligible fluctuations. Moreover, the RMSD values of the four ligand-protein complexes were below 2.5 Å, signifying that the complexes maintained their stability for the entire simulation period [41].

An evaluation of the SASA values of the top four complexes was performed to assess the surface variations caused by the interactions with the selected ligand molecules. A decrease in SASA value results in protein truncation, while an increase in SASA value results in the surface-area expansion of the protein [37]. In the CID_606333-7M1M, CID_553163-7M1M and CID_6421725-7M1M complexes, the SASA value initially decreased, whilst in the CID_569411-7M1M complex, the value increased initially. From 40–100 ns simulation time, the CID_553163-7M1M complex’s SASA was significantly greater than those of the other three complexes, indicating an expanded surface area of the complex [Figure 8b]. The SASA value was lower for CID_569411-7M1M complex than the other three complexes during the simulation time. At 80 ns, 75 ns, 70 ns, and 45 ns simulation time, the CID_606333-7M1M, CID_569411-7M1M, CID_553163-7M1M, and CID_6421725-7M1M complexes reached a steady state, and with only slight fluctuations they continued steadily for the remainder of the simulation period.

Protein complexes were assessed by using the Rg values to determine their compactness or flexibility. The Rg value on simulated protein complexes can be used as a measure of how flexible or firm they are, with a higher value indicating greater flexibility and a lower value suggesting greater firmness [33]. Specifically, for the CID_553163-7M1M, CID_6421725-7M1M, and CID_569411-7M1M complex, the Rg value was initially lower due to their increased flexibility, and for the CID_606333-7M1M complex, the Rg value was initially higher owing to their higher stiffness [Figure 8c]. With only minor fluctuations, the CID_606333-7M1M complex showed the highest range Rg value during the 5–35 ns simulation time frame, while the CID_6421725-7M1M complex displayed the lowest range Rg value between 75–100 ns simulation time frame.

A hydrogen-bonding analysis was performed on the docked complexes as hydrogen bonds are crucial for maintaining the firmness and integrity of the protein. A plenty of hydrogen bonds was formed throughout the simulation trajectory between the CID_606333-7M1M, CID_569411-7M1M, CID_553163-7M1M, and CID_6421725-7M1M complexes, which demonstrated a tight interaction between the top four ligand molecules and the 7M1M protein [Figure 8d]. A study of the RMSF of the top four ligands and 7M1M complexes was conducted in order to investigate the flexibility of 7M1M across the amino acid area. Nearly all amino acid residues except at the beginning of the top four docked complexes had RMSF values below 2.5 Å. Accordingly, the top four docked complexes had lower RMSF values, representing decreased flexibility as lower RMSF values correlate with the greater stability of the complex [Figure 8e] [37].

## 4. Discussion

A multi-drug-resistant, opportunistic, pathogenic bacteria, *Pseudomonas* sp. causes nosocomial infections in both plants and people [44]. The production of novel drugs or therapeutic targets is urgently required in light of the growth of multi-drug-resistant *Pseudomonas* species. Old molecules are also required in order to increase the effectiveness of current treatments or preventative measures [45,46]. *Pseudomonas* sp. has a remarkable capacity to amass a wide range of resistance through the formation of biofilms [47]. The appropriate formation of biofilms depends heavily on caseinolytic proteases (Clp) [14]. Therefore, CIp inhibitors would be useful for managing this disease.

Gram-positive bacteria called *Bacillus subtilis* produce a diverse variety of secondary metabolites that can be used to find new drug structures [48]. It was discovered that *Pseudomonas* sp. is resistant to the antibacterial effects of *Bacillus subtilis*. Similar results were discovered by Ozabor et al., who used the agar-well-diffusion method to uncover a 15mm inhibitory zone against *Pseudomonas aeruginosa* [49]. Additionally, it was established through additional research that *Bacillus subtilis* was effective against *Pseudomonas* sp. [50,51].

In the current investigation, we employed the CFS from *Bacillus subtilis* to prevent *Pseudomonas* sp. from forming biofilms. Here, it was discovered that *Pseudomonas* sp. biofilm was successfully inhibited by *Bacillus subtilis* CFS. Additionally, SEM images verified CFS’s anti-biofilm action on glass surfaces. These outcomes are consistent with earlier studies on the inhibition of pathogen biofilms by CFS bacterial cultures. Around 80% of *Vibrio harveyi* and 78% of *Pseudomonas aeruginosa*’s biofilm development was suppressed by *Bacillus licheniformis* [52]. Additionally, *Bacillus subtilis* KATMIRA1933 and *Bacillus amyloliquefaciens* B-1895 demonstrated antibacterial and anti-biofilm efficacy against *Acinetobacter* sp., both individually and in combination with polymyxin E [15].

Bioactive compounds from *Bacillus subtilis*’ volatile compounds were used in the docking interaction with *Pseudomonas* sp.’s biofilm-forming protein (PDB 7M1M). It was observed that the compound with the maximum binding energy to the protein that forms biofilms was 1-(9H-Fluoren-2-yl)-2-(1-phenyl-1H-tetrazol-5-ylsulfanyl)-ethanone. For their effectiveness in binding, oxalic acid, cyclohexylmethyl tridecyl ester, bis(pentamethylcyclotrisiloxy)tetramethyldisiloxane, and 2,2’-anhydro-1-arabinofuranosyluracil were chosen.

Moreover, from the top four compounds, 1-(9H-Fluoren-2-yl)-2-(1-phenyl-1H-tetrazol-5-ylsulfanyl)-ethanone and 2,2’-anhydro-1-arabinofuranosyluracil were screened as they obey ADME properties and the Lipinski drug rule properly. In previous studies, bioactive compounds from *Leonurus sibiricus* showed effectiveness against quorum-sensing proteins of *P. aeruginosa* through in vitro and in silico studies [53].

However, there are no other studies available where active bacterial compounds were used in in silico studies. Therefore, this is a recommendable approach to developing an antibiotic to obtain relief from this pathogenic multi-drug-resistant strain.

## 5. Conclusions

The usage of bacterial bioactive substances is becoming more and more popular. A Gram-positive bacterium, *Bacillus subtilis*, with probiotic properties exhibited antibacterial and anti-biofilm properties. This was supported by the outcomes of in silico interactions between the bioactive substances and the protein that forms biofilms. Furthermore, 1-(9H-Fluoren-2-yl)-2-(1-phenyl-1H-tetrazol-5-ylsulfanyl)-ethanone was identified as the most promising therapeutic candidate due to its drug-like characteristics and anti-biofilm efficacies against *Pseudomonas* sp. Additionally, this might offer a new opportunity for the development of antibiotics in the future.

## Figures and Tables

**Table 1:** Antagonistic activity of *Bacillus subtilis* against the selected bacteria. Different letters indicate significant differences between mean ± SD of replications (*n* = 3) at a *p* < 0.05 significance level.

Name of the Strains	Zone of Inhibition (mm)	Status
*Klebsiella pneumoniae*	07.00 ± 0.33 ^a^	No inhibitory activity (−)
*Pseudomonas* sp.	18.33 ± 1.00 ^c^	Moderate/average inhibition (++)
*Escherichia coli*	08.33 ± 0.57 ^ab^	No inhibitory activity (−)
*Staphylococcus aureus*	09.00 ± 1.00 ^a^	No inhibitory activity (−)

**Table 2:** Ligand No, PubChem CID of top 10 molecules based on docking-score binding affinity.

	Compounds	CID	Docking Score (kcal/mol)
1	1-(9H-Fluoren-2-yl)-2-(1-phenyl-1H-tetrazol-5-ylsulfanyl)- ethanone	606333	−7.0
2	Oxalic acid, cyclohexylmethyl tridecyl ester	6421725	−6.4
3	Bis(pentamethylcyclotrisiloxy)tetramethyldisiloxane	553163	−6.2
4	2,2’-Anhydro-1-arabinofuranosyluracil	569411	−6.0
5	Pyridine, 2,3,6-trimethyl-	15100	−5.8
6	D-Limonene	440917	−5.8
7	Azulene	9231	−5.8
8	Benzene, 1,3-bis(1,1-dimethylethyl)-	71343282	−5.8
9	Propionic acid, (3,6,7,8-tetrahydro-3,7-methano-2,4,6- trimethyl-2H-oxocin-7-yl)methyl ester	583617	−5.7
10	2,5-di-tert-Butyl-1,4-benzoquinone	17161	−5.6

**Table 3:** Pharmacological assessment of the screened hit ligand molecules.

Compounds Name and CID	MW g/mol	H. Ac	H. Do	Log Po/w	Log S	Number of Lipinski Violation	TPSA (Å^2^)	Human Intestinal Absorption)	BBB (+ve/−ve)
1-(9H-Fluoren-2-yl)-2-(1-phenyl-1H-tetrazol-5-ylsulfanyl)- ethanone; 606333	384.5	4	0	3.20	−5.70	Yes; 0 violation	85.97	High	−ve
Oxalic acid, cyclohexylmethyl tridecyl ester; 6421725	368.5	4	0	5.03	−6.61	Yes; 4 violations	52.60	High	−ve
Bis(pentamethylcyclotrisiloxy)tetramethyldisiloxane; 553163	579.2	9	0	6.36	−2.37	Yes; 2 violations	83.07	High	−ve
2,2’-Anhydro-1-arabinofuranosyluracil; 569411	330.29	7	1	2.11	−1.50	Yes; 0 violation	99.88	High	−ve

## Data Availability

The datasets used and/or analyzed during the current study are available from the corresponding author on reasonable request.

## Supplementary Materials

The following supporting information can be downloaded at: https://www.mdpi.com/article/10.3390/microorganisms10112105/s1, Table S1: List of bacterial strains used for the antagonistic test; Table S2: Ligand No, PubChem CID of the molecules identified through GC-MS based and their docking score; Table S3: Protein-Ligand interactions of Caseinolytic proteases (Clp) enzymes (PDB ID: 7M1M) with top 8 volatile compounds of *Bacillus subtilis*.
