# Supplementary material for: Cell-Free Supernatants (CFSs) from the Culture of Bacillus subtilis Inhibit Pseudomonas sp. Biofilm Formation
Source: Microorganisms. 2022 Oct 24;10(11):2105. doi: 10.3390/microorganisms10112105 (PMC9692604; doi:10.3390/microorganisms10112105)
Supplement: Supplementary file 1 [file microorganisms-10-02105-s001.zip › microorganisms-1945773-supplementary.pdf]

# Cell-Free Supernatants (CFSs) from the Culture of *Bacillus subtilis* Inhibit *Pseudomonas* sp. Biofilm Formation

**Shirmin Islam [1], Md. Liton Mahmud [1], Waleed H. Almalki [2,*], Suvro Biswas [1], Md. Ariful Islam [1], Md. Golam Mortuza [3], Mohammad Akbar Hossain [4], Md. Akhtar-E Ekram [1], Md. Salah Uddin [1], Shahriar Zaman [1] and Md. Abu Saleh [1,*]**

1 Microbiology Laboratory, Department of Genetic Engineering and Biotechnology, University of Rajshahi, Rajshahi 6205, Bangladesh
2 Department of Pharmacology and Toxicology, Faculty of Medicine, Umm Al-Qura University, Makkah 21955, Saudi Arabia
3 Department of Science and Humanities, Bangladesh Army International University of Science and Technology, Cumilla 3500, Bangladesh
4 Department of Pharmacology and Toxicology, Faculty of Medicine in Al-Qunfudah, Umm Al-Qura University, Makkah 21955, Saudi Arabia
* Correspondence: whmalki@uqu.edu.sa (W.H.A.); saleh@ru.ac.bd (M.A.S.)

**Table S1: List of bacterial strains used for the antagonistic test**

| Sl. no. | Name of the used bacterial strains |
|---|---|
| 01 | *Klebsiella pneumoniae* |
| 02 | *Pseudomonas* sp. |
| 03 | *Escherichia coli* |
| 04 | *Staphylococcus aureus* |

**Table S2: Ligand No, PubChem CID of the molecules identified through GC-MS based and their docking score**

| Compounds | CID | R.T | Area Percentage | Docking Score |
|---|---|---|---|---|
| 1. 1-Butanol, 3-methyl- | 31260 | 1.965 | 0.06 | -4.2 |
| 2. Disulfide, dimethyl | 12232 | 4.327 | 6.06 | -2.3 |
| 3. Butanoic acid, 2-methyl- | 8314 | 6.933 | 0.19 | -4.6 |
| 4. Oxime-, methoxy-phenyl-_ | 9602988 | 7.346 | 0.21 | -5.0 |
| 5. 2-Heptanone | 8051 | 7.531 | 0.67 | -4.9 |
| 6. Anisole | 7519 | 8.104 | 0.46 | -4.9 |
| 7. Hexanoic acid, methyl ester | 7824 | 8.25 | 0.08 | -4.9 |
| 8. 2-Heptanone, 4-methyl- | 94317 | 8.507 | 0.1 | -4.0 |
| 9. 1-(9H-Fluoren-2-yl)-2-(1-phenyl-1H-tetrazol-5-ylsulfanyl)-ethanone | 606333 | 8.755 | 0.15 | -7.0 |
| 10. Dimethyl trisulfide | 19310 | 8.821 | 0.86 | -2.6 |
| 11. Benzaldehyde | 240 | 9.045 | 0.18 | -4.8 |
| 12. Pentasulfide, dimethyl | 81772 | 9.184 | 8.51 | -2.4 |
| 13. 3-Cyclopentyl-1-propyne | 521007 | 9.411 | 14.45 | -5.4 |
| 14. Phenol, 2-chloro- | 7245 | 9.597 | 2.34 | -4.4 |
| 15. Pyridine, 2,3,6-trimethyl- | 15100 | 9.696 | 1.74 | -5.8 |
| 16. Propanoic acid, 2,2-dimethyl-, propyl ester | 138418 | 9.776 | 1.02 | -4.2 |
| 17. Pyrazine, trimethyl- | 26808 | 9.866 | 0.63 | -5.2 |
| 18. Diethylene glycol tert-butyl ether methyl ether | 104324 | 10.003 | 0.24 | -4.3 |
| 19. Benzene, 1,4-dichloro- | 4685 | 10.09 | 0.13 | -5.0 |
| 20. Butane, 1-[(1-methylethyl)thio]- | 522478 | 10.15 | 0.15 | -3.4 |
| 21. 5-Hydroxy-hex-2-enoic acid, methyl ester | 5369246 | 10.22 | 0.13 | -5.0 |
| 22. 5-Chloropentanoic acid, 2-ethylcyclohexyl ester | 544276 | 10.262 | 0.19 | -4.7 |

| | | | | |
|---|---|---|---|---|
| 23. 2-Ethyl-1-hexanol | 7720 | 10.319 | 0.18 | -4.8 |
| 24. D-Limonene | 440917 | 10.355 | 0.21 | -5.8 |
| 25. Propane, 2-(chloromethyl)-1,3-dimethoxy-2-methyl- | 542360 | 10.527 | 0.21 | -3.5 |
| 26. Oxirane, 2-[2-(benzyloxy)-1-(1-methoxy-1-methylethoxy)ethyl] | 552649 | 10.626 | 0.23 | -4.8 |
| 27. 2-Nonanone | 13187 | 10.773 | 0.11 | -5.0 |
| 28. Propionic acid, (3,6,7,8-tetrahydro-3,7-methano-2,4,6-trimethyl-2H-oxocin-7-yl)methyl ester | 583617 | 10.848 | 0.11 | -5.7 |
| 29. Pyrazine, 3-ethyl-2,5-dimethyl- | 25916 | 11.203 | 0.56 | -4.4 |
| 30. 1-Heptanol, 2,4-dimethyl-, | 101564277 | 11.28 | 0.1 | -5.4 |
| 31. o-Toluic acid, 2-ethylhexyl ester | 577168 | 11.363 | 0.36 | -5.5 |
| 32. 2-Nonanone | 13187 | 11.43 | 0.92 | -5.0 |
| 33. 2,5-Dihydroxybenzaldehyde, 2TMS derivative | 622536 | 11.476 | 1.27 | -4.0 |
| 34. 2,2'-Anhydro-1-arabinofuranosyluracil | 569411 | 11.55 | 0.12 | -6.0 |
| 35. 1-Heptanol, 2-propyl- | 24847 | 11.617 | 0.23 | -4.5 |
| 36. Nonanal | 31289 | 11.675 | 0.1 | -4.2 |
| 37. Succinic acid, 3,7-dimethyloct-6-en-1-yl pentyl ester | 91702362 | 11.748 | 0.19 | -4.1 |
| 38. Phorone | 10438 | 11.79 | 0.16 | -5.1 |
| 39. Phenylethyl Alcohol | 6054 | 11.873 | 0.37 | -5.1 |
| 40. Benzene, 1-chloro-4-methoxy- | 12167 | 11.99 | 0.29 | -4.2 |
| 41. Silane, dimethyl(dimethyl(dimethyl(2-isopropylphenoxy)silyloxy)silyloxy)(2-isopropylphenoxy)- | 91743286 | 12.015 | 0.19 | -3.3 |
| 42. Benzenemethanol, 4-methyl- | 11505 | 12.264 | 0.22 | -5.5 |
| 43. 2,6-Dodecadien-1-al | 6430759 | 12.387 | 0.1 | -5.2 |
| 44. (+)-3-Carene, 2-(acetylmethyl)- | 576614 | 12.546 | 0.2 | -5.4 |
| 45. Hexadecanal, 2-methyl- | 546976 | 12.634 | 0.06 | -5.1 |
| 46. 1-Methoxy-2-methyl-4-(methylthio)benzene | 592820 | 12.724 | 0.22 | -4.4 |

| | | | | |
|---|---|---|---|---|
| 47. 1,3-Propanediol, 2-butyl-2-ethyl- | 61038 | 12.83 | 0.14 | -4.6 |
| 48. Neophytadiene | 10446 | 12.943 | 1.36 | -4.6 |
| 49. n-Decyl methylphosphonofluoridate | 567104 | 13.104 | 0.35 | -4.6 |
| 50. Azulene | 9231 | 13.184 | 0.31 | -5.8 |
| 51. Fumaric acid, 2,5-dimethylphenyl nonyl ester | 91711256 | 13.28 | 0.22 | -5.4 |
| 52. 16-Methyl-heptadecane-1,2-diol, trimethylsilyl ether | 91742675 | 13.565 | 0.12 | -3.4 |
| 53. Tetrasulfide, dimethyl | 79828 | 13.677 | 2.24 | -2.3 |
| 54. Benzothiazole | 7222 | 13.834 | 1.51 | -5.1 |
| 55. Benzene, 1,3-bis(1,1-dimethylethyl)- | 71343282 | 14.086 | 0.75 | -5.8 |
| 56. 1,7-Di(3-ethylphenyl)-2,2,4,4,6,6-hexamethyl-1,3,5,7-tetraoxa-2,4,6-trisilaheptane | 91742720 | 14.203 | 0.05 | -7.1 |
| 57. Bicyclo[3.2.0]hepta-3,6-diene-1-carbonitrile | 575776 | 14.88 | 10.91 | -4.5 |
| 58. o-Methoxymandelic acid, 2TMS derivative | 530206 | 14.992 | 0.33 | -4.1 |
| 59. Pyrazine, 2,5-dimethyl-3-(3-methylbutyl)- | 519564 | 15.044 | 0.19 | -5.3 |
| 60. Trichloroacetic acid, 6-ethyl-3-octyl ester | 550155 | 15.105 | 0.05 | -4.9 |
| 61. 3-tert-Butyl-2-pyrazolin-5-one | 520421 | 15.169 | 0.21 | -5.2 |
| 62. E-10,13,13-Trimethyl-11-tetradecen-1-ol acetate | 5365074 | 15.565 | 0.05 | -4.5 |
| 63. 3,5-Dibutoxy-1,1,1,7,7,7-hexamethyl-3,5-bis(trimethylsiloxy)tetrasiloxane | 551995 | 15.885 | 0.71 | -3.4 |
| 64. Dodecanedioic acid, 2TBDMS derivative | 634058 | 15.745 | 0.08 | -4.2 |
| 65. 3-(2-Hydroxy-cyclopentylidene)-2-methyl-propionic acid | 5369050 | 15.824 | 0.13 | -5.2 |
| 66. Ethane, 2-chloro-1-ethoxy-1-methoxy- | 548381 | 15.94 | 0.08 | -3.4 |
| 67. Dodecanedioic acid, 2TBDMS derivative | 634058 | 15.968 | 0.09 | -3.5 |
| 68. 16-Methyl-heptadecane-1,2-diol, trimethylsilyl ether | 91742675 | 16.005 | 0.06 | -4.3 |
| 69. 3,5-Dibutoxy-1,1,1,7,7,7-hexamethyl-3,5-bis(trimethylsiloxy)tetrasiloxane | 551995 | 16.105 | 0.2 | -4.2 |
| 70. 1-Heptacosanol | 74822 | 16.175 | 0.05 | -4.7 |

| | | | | |
|---|---|---|---|---|
| 71. Tetradecane | 12389 | 16.287 | 0.12 | -5.0 |
| 72. Dodecanedioic acid, 2TBDMS derivative | 634058 | 16.388 | 0.11 | -4.1 |
| 73. Carbonic acid, 2-ethylhexyl heptadecyl ester | 91693166 | 16.45 | 0.06 | -5.0 |
| 74. 2-Piperidinone, N-[4-bromo-n-butyl]- | 536377 | 16.58 | 0.05 | -4.4 |
| 75. 2,5-di-tert-Butyl-1,4-benzoquinone | 17161 | 17.337 | 0.19 | -5.6 |
| 76. 2-Dodecen-1-yl(-)succinic anhydride | 5362708 | 17.524 | 0.13 | -5.2 |
| 77. 2-Tridecanone | 11622 | 17.593 | 0.33 | -4.3 |
| 78. Phenol, 3,5-bis(1,1-dimethylethyl)- | 5614 | 17.777 | 4.66 | -4.2 |
| 79. N1,N1,N4-Tris(tert-butyldimethylsilyl)succinamide | 91744783 | 17.98 | 0.29 | -4.3 |
| 80. Bis(pentamethylcyclotrisiloxy)tetramethyldisiloxane | 553163 | 18.062 | 0.07 | -6.2 |
| 81. 1,4-Methanobenzocyclodecene, 1,2,3,4,4a,5,8,9,12,12a-decahydro- | 556414 | 19.992 | 0.04 | -4.3 |
| 82. 1,1,1,3,5,7,9,11,11,11-Decamethyl-5-(trimethylsiloxy)hexasiloxane | 6329082 | 20.093 | 0.18 | -4.8 |
| 83. Oxalic acid, cyclohexylmethyl tridecyl ester | 6421725 | 20.658 | 1.33 | -6.4 |
| 84. 7,9-Di-tert-butyl-1-oxaspiro(4,5)deca-6,9-diene-2,8-dione | 545303 | 24.3 | 0.25 | -3.3 |
| 85. Tetracosamethyl-cyclododecasiloxane | 167767 | 36.94 | 0.85 | -3.6 |

**Table S3:** Protein-Ligand interactions of Caseinolytic proteases (Clp)) enzymes (PDB ID: 7M1M) with top 8 volatile compounds of *Bacillus subtilis*

| **Compound Name** | **Hydrogen bond** | | **Hydrophobic bond** | |
|---|---|---|---|---|
| | Residues | Distance (A°) | Residues | Distance (A°) |
| 1-(9H-Fluoren-2-yl)-2-(1-phenyl-1H-tetrazol-5-ylsulfanyl)-ethanone | | | Met-41<br>Tyr-22 | 5.8<br>4.40 |

| | | | | |
|---|---|---|---|---|
| | | | Tyr-22 | 4.04 |
| Oxalic acid, cyclohexylmethyl tridecyl ester | Thr-22<br>Thr-22 | 4.46<br>5.15 | | |
| Bis(pentamethylcyclotrisiloxy)tetramethyldisiloxane | Gln-48 | 5.27 | Tyr-22<br>Tyr-22<br>Tyr-22<br>Tyr-22<br>Tyr-22<br>Val-5 | 4.19<br>4.48<br>4.57<br>5.36<br>4.78 |
| 2,2'-Anhydro-1-arabinofuranosyluracil | Val-34<br>Val-34 | 3.17<br>4.24 | Met-41<br>Tyr-22 | 5.59<br>4.21 |
